# Thinking Outside the Ischemia Box: Advancements in the Use of Multiple Sclerosis Drugs in Ischemic Stroke

**DOI:** 10.3390/jcm10040630

**Published:** 2021-02-07

**Authors:** Athina-Maria Aloizou, Vasileios Siokas, Georgia Pateraki, Ioannis Liampas, Christos Bakirtzis, Zisis Tsouris, George Lazopoulos, Daniela Calina, Anca Oana Docea, Aristidis Tsatsakis, Dimitrios P. Bogdanos, Efthimios Dardiotis

**Affiliations:** 1 Department of Neurology, University Hospital of Larissa, Faculty of Medicine, School of Health Sciences, University of Thessaly, 41110 Larissa, Greece; aaloizou@med.uth.gr (A.-M.A.); vsiokas@med.uth.gr (V.S.); georginapat08@gmail.com (G.P.); liampasioannes@gmail.com (I.L.); zitsouri@med.uth.gr (Z.T.); 2 Multiple Sclerosis Center, B’ Department of Neurology, AHEPA University Hospital, Aristotle University of Thessaloniki, 54636 Thessaloniki, Greece; bakirtzischristos@yahoo.gr; 3 Department of Cardiothoracic Surgery, University General Hospital of Heraklion, Medical School, University of Crete, 71003 Heraklion, Greece; lazopoulosg@ath.forthnet.gr; 4 Department of Clinical Pharmacy, University of Medicine and Pharmacy of Craiova, 200349 Craiova, Romania; calinadaniela@gmail.com; 5 Department of Toxicology, University of Medicine and Pharmacy of Craiova, 200349 Craiova, Romania; daoana00@gmail.com; 6 Laboratory of Toxicology, School of Medicine, University of Crete, 71003 Heraklion, Greece; tsatsaka@uoc.gr; 7 Department of Rheumatology and Clinical Immunology, Faculty of Medicine, School of Health Sciences, University of Thessaly, 41110 Larissa, Greece; bogdanos@uth.gr

**Keywords:** ischemic stroke, multiple sclerosis, natalizumab, fingolimod

## Abstract

Ischemic stroke (IS) is a major cause of death and disability, despite early intervention. Thrombo-inflammation, the inflammatory process triggered by ischemia, is a concept that ties IS with multiple sclerosis (MS), under the wider ‘umbrella’ of neuroinflammation, i.e., the inflammation of the nervous tissue. Drawing from this, numerous studies have explored the potential of MS disease-modifying drugs in the setting of IS. In this review, we present the available studies and discuss their potential in ameliorating IS outcomes. Based on our search, the vast majority of the studies have been conducted on animals, yielding mostly positive results. Two clinical trials involving natalizumab showed that it does not confer any benefits, but four human studies regarding fingolimod have showcased its potential in improving recovery prospects. However, concerns on safety and other issues are raised, and basic questions still need to be answered.

## 1. Introduction

Ischemic stroke (IS) accounts for approximately 87% of all stroke cases, and is a major cause of death and permanent disability in developed countries [1,2]. In ischemic stroke, an in situ thrombus formation or the migration of a blood clot from the periphery blocks the blood flow in a cerebral artery. As a consequence of this, a percentage of cells mainly catered for by the blocked artery die, and create the main ischemic core. The surrounding, gravely hypoperfused tissue can still be rescued before it also becomes part of the ischemic core. This region is called the ‘penumbra’ and its salvation is, in principle, the target of all therapeutic interventions [3]. The main treatment options in IS are intravenous alteplase/recombinant tissue plasminogen activator (rTPA) and endovascular thrombectomy [4], but even when these are applied in a timely manner, many individuals do not regain their functional status [3]. One of the hypothesized causes is the perfusion failure of the microvasculature downstream of the occluded vessel, which leads to a progressive increase in infarct volume [5].

In classic pathophysiology terms, cerebral ischemia is divided into three stages; in the first stage, the blood-brain barrier (BBB) is disrupted and leukocytes enter the ischemic area, while in the second macrophages also infiltrate, and astrocytes multiply and activate [6]. The presence of these inflammatory cells in ischemia, and the evidence of increased inflammatory components in IS, has led to the description of the “thrombo-inflammation” concept; ischemia is now known to trigger a cascade of sterile inflammatory processes, which aggravate ischemic damage. In greater detail, cerebral ischemia leads to the upregulation of cell adhesion molecules, which facilitate leukocyte influx into thecentral nervous system (CNS). Neutrophils, monocytes, and lymphocytes infiltrate the parenchyma in succession, driving brain damage via numerous pathways [7]. As such, the compromised BBB and the subsequent neuroinflammation, i.e., the inflammation of nervous tissue, lead to additional cell death and thus lower recovery prospects [8]. Additionally, the reperfusion attempts, especially when delayed, may also aggravate inflammation and hinder recuperation, leading to the so-called “reperfusion injury”; the reactive hyperemia disrupts cerebral autoregulation and BBB junctions, further facilitating white cell influx and inflammatory processes [9].

In this regard, neuroinflammation is an important common element in IS and multiple sclerosis (MS), despite it having different origins in the two entities (ischemia/thromboinflammation vs. autoimmunity). The common elements mostly regard glia cell activation and immune cell migration into the CNS [10,11]. MS is the commonest autoimmune disease of the CNS, characterized by neuroinflammation, demyelination and, subsequently, neurodegeneration, which leads to the progressive accumulation of disability in patients [12,13]. Given the attention inflammation and immune activation has been gathering in the context of IS [14], and the fact that MS treatment modalities targeting the CNS are usually immunosuppressive/immunomodulatory [15,16], research is being conducted on whether substances used in MS can ameliorate IS parameters and recovery. In fact, studies in the respective experimental models have shown that treatment modalities fitted to MS may indeed be beneficial in IS [11,17].

This review aims to present the studies exploring the effectiveness of MS disease-modifying drugs (DMDs) in IS, with a focus onhuman studies, or recent animal studies where no human studies were available. Additionally, we aim to discuss the overall potential of these treatments and the matters that should be handled with caution.

## 2. Methods

Our main focus was gathering the clinical trials/human studies available, and this review contains two sections, one for DMDs with clinical evidence, and one for DMDs with only preclinical evidence. As such, for the ‘clinical’ part of our review we searched the PubMed database with “ischemic stroke” and the name of each DMD as keywords, in particular “natalizumab”, “fingolimod”, “interferon-beta”, “glatiramer acetate” and “copaxone”, “teriflunomide”, and “dimethyl fumarate”. Relevant manuscripts were first scanned by title and then abstract. Those fulfilling the inclusion criteria were read in full-text, and their reference lists were scanned for any omitted items. Additionally, we searched the clinicaltrials.gov (accessed on 25 November 2020) website, for any ongoing trials on this matter.

Inclusion criteria were: (a) Studies on humans >18 years of age; (b) Existence of a control group; (c) Patients were diagnosed with ischemic stroke.

Exclusion criteria were: (a) Reviews, commentaries, and case-reports; (b) Studies on animals; (c) Studies not describing the administration protocol for the DMD; (d) Studies involving hemorrhagic stroke or intracranial hemorrhage. For the preclinical part of our review, we retrieved the most recent preclinical animal studies, or meta-analyses of animal studies on the use of MS DMDs on animal models of IS, which emerged through the search for clinical studies, and were thus excluded from the first part. Similarly, selected reviews, although excluded from the first part’s algorithm, were also read and assessed for potentially omitted studies.

## 3. Results of Multiple Sclerosis DMDs

The search algorithm for clinical studies yielded the following results:

- Searching for “natalizumab” and “ischemic stroke”, eight results came up, published from 2014 until 2020, and all of their abstracts were assessed. Six were rejected as reviews or animal studies, and two fulfilled the inclusion criteria.
- Searching for “fingolimod” and“ischemic stroke”, 42 results came up, published from 2009 to 2020. The abstracts from 36 of these were assessed. Eight were excluded as reviews or involving hemorrhage, and from the rest, four fulfilled the inclusion criteria, and one described the rationale of a trial currently being conducted.
- Searching for “interferon-beta” and “ischemic stroke”, 64 results came up, published from 1995 to 2020. The abstracts from 19 of those were assessed, and all failed to fulfill the inclusion criteria.
- Searching for “glatiramer acetate” and “ischemic stroke”, nine results came up, published from 2007 to 2020. The abstracts of six of those were assessed, and all failed to fulfill the inclusion criteria. The same results were yielded when “copolymer-1” was used instead of “glatiramer acetate”.
- Searching for “teriflunomide” and “ischemic stroke”, two results came up, published from 2016 to 2020. The abstract of one of these was assessed and deemed not fit for inclusion.
- Searching for “dimethyl fumarate” and “ischemic stroke”, 11 results came up, published from 2015 to 2020. The abstracts of 10 of these were assessed, and all failed to fulfill the inclusion criteria.

**Figure 1:**
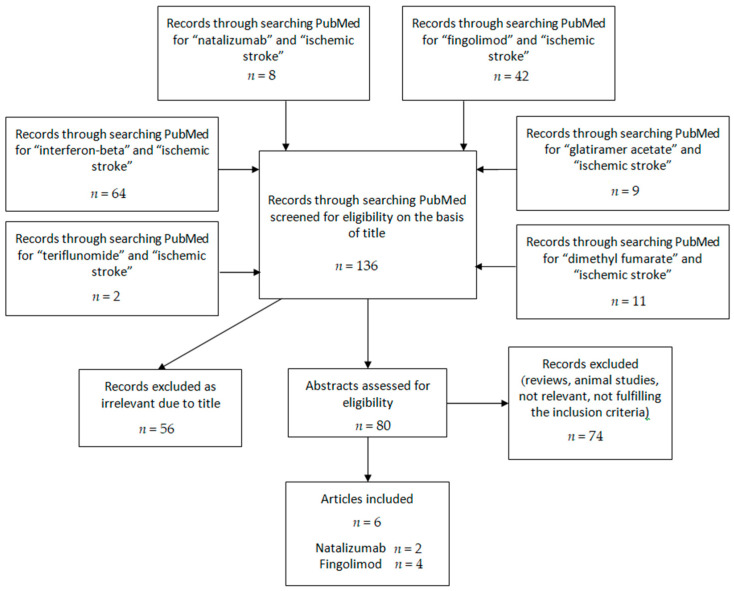
Flowchart for the search algorithm’s results.

**Figure 2:**
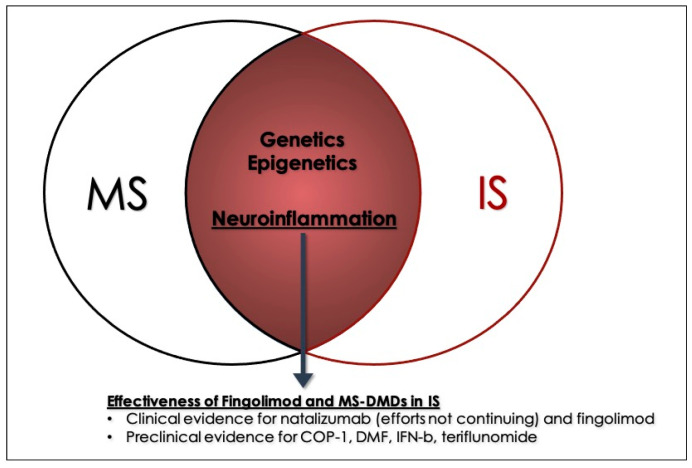
Neuroinflammation, the overlap between Multiple Sclerosis (MS) and Ischemic Stroke (IS) gives way to the application of MS-disease-modifying drugs to be applied in cerebral ischemia.

Table 1 provides a brief overview of the mechanism of actions of the DMDs included, while Table 2 and Table 3 provide a concise and detailed summary, respectively, of the studies involving humans involving the use of MS DMDs in IS. The search algorithm’s results can be seen in the flowchart of Figure 1, while a graphical abstract can be seen in Figure 2.

### 3.1. DMDs with Clinical Evidence

#### 3.1.1. Natalizumab

Natalizumab (Tysabri, Biogen, Cambridge, MA, USA) is a selective inhibitor of adhesion molecules and binds to the α4 subunit of human integrins, which is strongly expressed on the surface of all leukocytes except neutrophils. Specifically, natalizumab binds to α4β1 integrin, blocking its interaction with its related receptor. Blocking the molecular interactions of α4β1 with its targets reduces the inflammatory activity present in the brain in MS and inhibits the further recruitment of immune cells into inflamed tissue, thus reducing the formation or enlargement of the lesions [18]. The blocking of white cell influx into the CNS can also be beneficial in the setting of IS, since at the first stages of cerebral ischemia, the disruption of the BBB and the increase of cell adhesion molecules allow the migration of leukocytes into the brain parenchyma, and induceneuroinflammation.

Until 2015, seven animal studies exploring the effects of natalizumab on IS had been published [17]. The majority of them reported a reduction in infarct size with natalizumab administration [32,33,34,35] and an improvement in recovery outcomes [34,35]. One study assessed the effects of natalizumab before and after the stroke, in both TMCAO (temporary middle cerebral artery occlusion) and PMCAO (permanent MCAO) models, but failed to detect any improvement in stroke outcome [36]. Finally, a multicenter high-quality study attempting to replicate the conditions of an actual clinical trial, reported that natalizumab conferred positive results only in the setting of PMCAO at a distal point, which created small ischemic lesions, and not in the setting of TMCAO, which produced cortical and subcortical lesions to a bigger brain area. Additionally, the significant improvements were only noted when the results from all the centers were pooled together, and not when each center was individually assessed. Those findings supported the notion that natalizumab’s efficacy may depend on infarct size and location, and may thus not be unanimously effective [37]. Following these findings, one more recent animal study has been conducted, and reported that neither early nor delayed (1h vs. 48h post-stroke) natalizumab administration reduced infarct volume and neurological deficits; only early administration temporarily improved neurological function, but this improvement did not last (noted at 24h, disappeared by the fifth day). In fact, the applied IS model also created small cortical lesions but, unlike the findings of the previous study, no amelioration was shown whatsoever [18].

The initial positive results of the animal studies led to two human clinical trials. The results of the first ACTION trial (randomized, placebo-controlled, double-blinded, phase 2, NCT (National Clinical Trial) Identifier: NCT01955707) were published in 2017. In this study, acute IS patients received 300mg of IV (intravenous) natalizumab (79 patients) or placebo (82 patients) up until 9h after IS occurrence. The adverse events and deaths were similar between the two groups, although two patients in the treated group died of reasons attributed to the medication. Assessment at day 1, day 5 and day 30 reported no significant differences in infarct volume and neurological deficits. However, a greater percentage of treated patients had better function at day 90, as assessed by Barthel Index (BI), and higher MoCA (Montreal Cognitive Assessment Test) scores [26].

Based on these findings, and the fact that in ACTION patients that went on to receive higher doses of the medication had better results, the investigators carried out the ACTION II trial (NCT Identifier: NCT02730455), whose results were published very recently. In this study, acute IS patients received either 300mg (88 patients) or 600mg (89 patients) of IV natalizumab, or placebo (90 patients), and were stratified based on the time from their last known normal state (less than 9h or more than 9h but less than 24h). No differences in adverse events or deaths were noted between the three groups, but patients receiving natalizumab were less likely to present better outcomes in either treatment branch. The results remained negative for a beneficial effect on outcome even when the data of both ACTION trials were pooled together, and in view of this the trial was discontinued [27].

Given the fact that the no-benefit results of ACTION II were consistent across treatment branches, endpoints and other subgroup classifications, and that the positive results of ACTION were only shown in a specific setting and were mild, it is most likely that natalizumab has a null effect on the outcome of IS.

#### 3.1.2. Fingolimod

Fingolimod, or FTY720, (Gilenya, Novartis, Basel, Switzerland) is a sphingosine-1-phosphate (S1P) receptor agonist that was approved for RRMS in 2010, as the first oral agent of its kind [38]. S1P is an intracellular messenger that takes part in numerous processes, such as cellular differentiation, immune reactions, and B and T cell circulation. Through functional antagonism of the S1P-1 receptor, fingolimod influences the trafficking of CD4+ T cells and the migration of lymphocytes and, as a consequence, combats neuroinflammation [19]. In a similar way to natalizumab, fingolimod could prevent leukocyte aggregation into the CNS following ischemia onset, and thus combatthrombo-inflammation.

Regarding IS, fingolimod is the MS-DMD that has probably been examined most often, and seems to aid in a plethora of parameters via a wide variety of actions [39]. A very recent meta-analysis on several animal studies of IS reported that fingolimod considerably reduced infarct size, attenuated deficits and ameliorated neurobehavioral recovery, with post-ischemia administration yielding better results [40]. This is particularly encouraging regarding the way these drugs could be administered in a realistic setting, i.e., after the onset of ischemia, and not as prophylaxis.

The first human pilot trial was conducted by Fu et al. in 2014. Twenty-two patients with anterior circulation occlusion and an onset of more than 4.5 h, thus not eligible for thrombolysis, were randomized to either receive standard treatmentor standard treatment and 0.5mg of oralfingolimod for three consecutive days. As evaluated by NIHSS scores (National Institutes of Health Stroke Scale), patients receiving fingolimod showed milder deficits and a speedier recovery, with this effect being especially evident in the first week. Lesion increase was also smaller in the treated group. An additional finding was that patients with total or partial anterior occlusion profited much more than patients with a lacunar infarct, and that the benefit from this intervention persisted after three months, as assessed by three different scales, showing that fingolimod positively influences long-term recovery [28].

Fingolimod has also been shown to be effective when combined with rTPA. The first study of this kind enrolled 47 patients eligible for thrombolysis, with 22 of these also randomized to receive 0.5mg of oral fingolimod for three consecutive days. Patients receiving the treatment showed smaller lesion volumes and restricted lesion growth within the first week, while their NIHSS scores were significantly lower on day 1. Additionally, they presented better recovery when assessed at three months. Regarding hemorrhagic transformation, fingolimod also reduced hemorrhage size [29]. Liantao et al. (2019) further explored the concomitant administration of 0.5mg of oral fingolimod for three days, and rTPA, by randomizing 45 patients to receive both and 45 to receive just rTPA. They reported that no difference in BI, mRS (modified Rankin Scale) and NIHSS scores was evident at a two-week assessment, but upon assessing the patients after three months, significant differences were noted (NIHSS and mRS significantly lower than in the control group, BI index significantly higher). Additionally, fingolimod contributed to reducing infarct volumes, assessed after a week [31].

Tian et al. (2018) addressed the efficacy of fingolimod in the setting of delayed rTPA treatment. They randomized 46 patients with internal carotid artery or middle cerebral artery proximal occlusion into either receiving both, or just rTPA. Within the first day, the group that received the treatment showed reduced infarct growth and faster improvement. The three-month assessment showed sustained amelioration in recovery. Finally, fingolimod significantly ameliorated anterograde and retrograde reperfusion, by preventing collateral circulation failure [30].

As it is evident by the results of the aforementioned studies, fingolimod seems to be a promising addition to the standard treatment of IS, preventing reperfusion injury, hemorrhagic transformation, and BBB disruption, while simultaneously reducing infarct size and improving prognosis. Following the encouraging results of the fingolimod-rTPA pairing, a clinical trial on the potential combination of fingolimod with rTPA and mechanical thrombectomy (bridging therapy) is being conducted (Fingolimod with Alteplase Bridging with Mechanical Thrombectomy in Acute Ischemic Stroke (FAMTAIS), NCT Identifier: NCT02956200). Ninety-eight patients eligible for bridging therapy have been randomized, and the study will assess penumbra tissue salvaging, infarct growth restriction, clinical improvement, and hemorrhagic transformation [41]. Additionally, another clinical trial (NCT Identifier: NCT04629872) aims to assess its efficacy in conjunction with endovascular treatments of IS, and is currently in the recruiting phase. Hopefully, even more trials will follow in the near future.

### 3.2. DMDs with Preclinical Evidence

#### 3.2.1. Interferon Beta

Interferon beta (IFN-b) (Rebif, Merck Serono, Darmstadt, Germany, and Avonex, Genesis Pharma, Athens, Greece) is an endogenous cytokine of the interferon family, which is involved in immune reactions against viral infections, stimulating numerous pathways that promote physiological cellular death or survival [20]. IFN-b responds to pathogenic injury and activates a wide array of cytokines; it has been used as an effective treatment for RRMS, thought to act on inducing anti-inflammatory pathways, reducing cell trafficking via the BBB, and promoting neuronal repair [21]. Due to its versatile anti-inflammatory and cell-regulating properties, it is hypothesized to be potentially beneficial also in the setting of IS [20].

Until 2017, six animal studies on the use of IFN-b in IS had been conducted [17]. The studies employed mice, rats, or rabbits in various stroke models and the majority of these showed a positive effect regarding infarct volume [42,43,44] (additional source: poster of Veldhuis et al. at the 2002 ISMRM-10th scientific meeting and exhibition in Honolulu, “Delayed treatment with interferon-beta protects against ischemic stroke”, available online) and neurological function regain [44], while one did not show a protective effect and raised safety concerns for the substance [45]. Very recently, Kuo et al. (2020) showed that IFN-b reduced the rates of mortality, of hemorrhagic transformation, and of adverse events induced by delayed rTPA administration, which has been associated with aggravated brain injury in a murine IS model [46].

Currently one clinical trial (Safety Study of Interferon Beta 1a for Acute Stroke, NCT Identifier: NCT00097318) is being conducted. This randomized, double-blind, placebo-controlled sequential dose escalation, phase 1 trial will enroll patients with acute IS and monitor them for 28 days following the stroke, assessing them for side-effects in order to establish the safety of this treatment in IS, before efficacy studies commence.

#### 3.2.2. Glatiramer Acetate

Glatiramer acetate or Copolymer-1 (Cop-1) (Copaxone, Teva, Petah Tikva, Israel) is a synthetic peptide that aids in the development of protective autoimmunity and has a plethora of anti-inflammatory properties; it activates and promotes the immunomodulatory functions of helper and regulatory T cells, and shifts the production of cytokines to an anti-inflammatory phenotype [22]. Additionally, it promotes neurogenesis, by inducing the formation of pro-neurogenic factors, such as BDNF (brain derived neurotrophic factor) and NT-3 (neurotrophin-3) [23,47]. The prospect of neuronal regeneration, which Cop-1 seems to support, is particularly important for ischemia, where neurons die and recovery prospects are thus diminished. All its beneficial properties, plus an attractive safety profile, have rendered glatiramer acetate a first-line DMD for MS [47] and have inspired a series of experiments on its possible use in IS.

In short, until 2015, two studies applying a model of TMCAO in rats supported that Cop-1 had potential in IS treatment, promoting neurogenesis and functional recovery, and reducing infarct volume in acute (seven days post-stroke) and chronic (60 days post-stroke) phases [48,49]. However, two other animal studies reported no beneficial effect of Cop-1 on IS in the first week [50,51]. The discrepancies between the studies have been attributed to the different time windows applied, as those that did not show a significant effect mostly assessed the mice within seven days of the stroke, while the study showing the highest effect of Cop-1 [48] reported that this effect was stronger after the first week. Additionally, one of the studies, demonstrating no results, did in fact report increased neurogenesis on day 7, but only on a model of PMCAO [50]. The studies were also based on different dosages and animal strains, all adding to the conflicting results.

Two recent studies came to tip the scales towards the beneficial effect of Cop-1 on IS. Cruz et al. delved into the pro-neurogenetic potential of the choroid plexus (CP), in mice assessed 14 days after TMCAO. Cop-1 significantly reduced neurological deficits, and increased neurogenesis in the “typical” neurogenetic sites (lateral ventricle walls/the subventricular zone and the sub-granular hippocampal zone), and the production of pro-neurogenetic factors and cytokines in the CP, the level of which was associated with the noted neurogenesis [52]. Mangin et al. (2019) took their research one step further, by bringing diabetes mellitus into the picture. This is one of the biggest IS risk factors, and diabetic patients usually have worse outcomes after a stroke, including increased incidence of post-stroke dementia [48]. In this study, diabetic mice were subjected to PMCAO and received Cop-1 for either 3 or 7 days after the stroke. One week after the stroke, treated mice showed reduced infarct volume and levels of proinflammatory molecules, and increased neurogenesis. The mice were assessed until six weeks after the stroke, and treated mice showed accelerated recovery ratesalready from day 3, and lower rates of long-term memory impairment [53].

Given these overall encouraging results in animal studies, we believe it is only a matter of time before Cop-1 is used in a human study, to address its safety and efficacy in ameliorating post-stroke recovery.

#### 3.2.3. Teriflunomide

Teriflunomide (Aubagio, Sanofi-Aventis, Paris, France) is a DMD approved for the treatment of relapsing-remitting MS (RRMS) [54]. It selectively inhibits dihydro-orotate dehydrogenase, a mitochondrial enzyme implicated in pyrimidine synthesis, and thus hinders the proliferation of activated B and T lymphocytes, and their consequent CNS infiltration. This ameliorates disease symptoms and reduces axonal loss, without severely suppressing the immune system [24].

In this sense, the reduction of these white cells and the consequent prevention of CNS migration can be beneficial in several entities. Aside from MS, a series of recent in vitro and in vivo (animal) studies have shown that it may also hold potential in neurological entities such as HIV-induced neurocognitive impairment [55], Batten disease, a lysosomal storage disease [56], and traumatic brain injury [57]. On the same note, only one recent study on IS and teriflunomide exists, performed on mice. The mouse-TMCAO model was used, assessing mice receiving teriflunomide and mice receiving only vehicle molecules three days after the “stroke”. Teriflunomide significantly reduced activated microglia around the infarct area, inhibited the induction of IL-10 and COX-2 inflammatory cytokines, promoted neurogenesis, and aided in the recovery of proper BBB function, by increasing growth factor PDGFB production and pericyte coverage of endothelial cells [58].

Until now, no trials with teriflunomide in stroke are ongoing and this is to be expected, given that there is only one animal study addressing its efficacy in IS. Regardless, it will be interesting to assess this in the future, considering that, unlike other regimens, this DMD does not cause severe immunosuppression, an important advantage, since stroke patients are already at higher risk of infections [59].

#### 3.2.4. Dimethyl Fumarate

Dimethyl fumarate (DMF) (Tecfidera, Biogen, Cambridge, MA, USA), a derivative of fumaric acid, has been used as a first-line agent for MS, as an anti-oxidant. It changes the composition of leukocyte subgroups, shifting their phenotype to anti-inflammatory, and induces T-cell apoptosis. DMF and its metabolite monomethyl-fumarate (MMF) also impair leukocyte permeation through the BBB, by downregulating adhesion molecules on the surface of vascular endothelium cells [25].

It is important to note, that oxidative stress (OS) is an important player in ischemia-induced damage, promoting neuroinflammation and leukocyte migration in the CNS [60]. The cellular defense mechanisms against OS are largely orchestrated by Nrf2 (nuclear factor erythroid 2-related factor 2), which regulates the expression of numerous detoxification genes [61], and DMF has been shown to act via Nrf2-dependent pathways [62]. As such, DMF could carry considerably potential regarding cerebral ischemia, via both leukocyte-migration blocking and antioxidative properties.

Regarding IS, DMF has only recently started to attract attention in preclinical studies. Clausen et al. (2017) applied a PMCAO model in mice, administering 20mg/kg of IV MMF and assessing the mice for 48h. Mice receiving the agent had reduced edema volume, though not reduced infarct size, and an overall better function. At the molecular level, MMF increased the levels of the anti-inflammatory IL-10 and Nrf2, and reduced those of the pro-inflammatory IL-12p70 [63]. Earlier studies with TMCAO models partially replicated their results; Kunze et al. (2015) also showed reduced edema but not infarct growth [64], while Yao et al. (2016) and Lin et al. (2016) showed improved recovery, and a reduced infarct size [65,66].

More recently, Liu et al. (2019) reported that pretreating mice before the “stroke” with DMF led to improved recovery, and reduced infarct growth and brain edema. However, this was not found in mice not expressing Nrf2, validating the association between DMF, Nrf2 and detoxification capability [67]. Safari et al. (2019) presented congruent results, with DMF pretreatment significantly reducing infarct size and increasing Nrf2 levels, while Nrf2 levels negatively correlated with infarct size [68]. Finally, Hou et al. (2020) published the results of their study, which drew data from a previous clinical trial (NCT Identifier: 03519828), on post-stroke cognitive impairment (PSCI), and then explored the possible protective effect of DMF in an animal IS model. The researchers reported that patients with PSCI showed reduced antioxidative capacity, while rats that received DMF showed better neurocognitive performance than the MCAO rats [69].

Conclusively, the overall results suggest that DMF/MMF in IS are promising, but the lack of human studies forbids any further speculation and, as such, more research in this direction is needed.

## 4. Discussion

The overlap between MS and IS contains several elements, with neuroinflammation and leukocyte migration into the CNS being two of the most prominent. Many of the first-line agents of MS have been explored as potential neuroprotective substances in cerebral ischemia, though only two have been involved in studies on human subjects, namely natalizumab and fingolimod. Natalizumab, due to a lack of positive results in two trials, is unlikely to be explored further, and as such fingolimod remains as the main focus of future research. The discovery of S1P agonists opened up an interesting new field in pharmacology, and fingolimod is now a very important weapon in our arsenal against MS [19]. As was discussed above, it might eventually join the strategies against IS, but before that point is reached some issues must be addressed for this drug, and for the interventions as a whole.

Firstly, the reasons behind natalizumab’s “failure” could be a valuable lesson for clinical trials in the future. The preclinical studies that led to the clinical trial conception had yielded conflicting results, and maybe the evidence showing a positive effect was weaker than that with a negative/null effect. In greater detail, the study by Llovera et al. (2015) [37], which most closely replicated the conditions of a clinical trial and was considered of great quality, the study by Langhauser et al. (2014) [36], and the most recent study by Drieu et al. (2020) [18] were all leaning towards natalizumab not being effective in IS, or being only conditionally effective. The latter employed a stroke model which most closely resembles human conditions (thrombin model), while the other two applied both the TMCAO and PMCAO models. As researchhas shown, the model used considerably influences the inflammatory response that occurs [70]. The TMCAO model, for example, which was mainly used in the studies that laid the groundwork for the clinical trials, seems to recreate the occurrence of secondary microthrombosis and neuroinflammation better. However, this insinuates that the negative results that were replicated in the actual clinical trials came from the models that did not produce the same extent of inflammation. This could mean that neuroinflammation may not be omnipresent in IS, and therefore only some patients will benefit from an immunomodulatory intervention. Additionally, all the “classic” reasons for reduced translatability from animal to human studies may also be at play [71], though the study by Llovera et al. was specifically designed to address and overcome the majority of these, so its results are possibly more accurate than others. It is further possible that more endeavors that produced negative results were held back from publication, in a case of “positive publication bias” [72]. Finally, none of the studies used comorbid animals, and it has been shown that the use of healthy animals tends to overestimate the effectiveness of an intervention in stroke research [73].

In the case of fingolimod, the recent meta-analysis by Dang et al. (2020) also evaluated the quality of the available preclinical literature, via the CAMARADES (Collaborative Approach to Meta Analysis and Review of Animal Data from Experimental Studies) checklist [40]. Per their results, almost two thirds of the studies used randomization, half of the studies were blinded regarding the outcome, and one third were blinded regarding the allocation. All but one study used anesthetic substances that possess no neuroprotective properties. One third of the studies also reported conflicts of interest. As such, most studies were of moderate quality. It is interesting to note that the majority of studies employed a TMCAO model to produce positive results, which is similar to natalizumab. The three studies that employed a permanent occlusion model all reported positive results [74,75,76] in that setting too, so it is possible that permanent occlusion models can more accurately “predict” whether a regimen will be effective in humans or not.

Fingolimod has been released as an oral agent. However, its gastrointestinal absorption rate is slow, and since, in IS, time is of the essence, parenteral administration might aid in achieving higher concentrations more quickly. As animal models showcased, IV or intraperitoneal injections led to better results, as did earlier administration [40]. Currently, novel S1P receptor modulators, with more favorable pharmacokinetic and safety profiles are being developed and tested [19], and some of these might also prove better for IS, but that remains to be seen. As of now, investigators have administered fingolimod orally in the available IS studies, and no direct comparison of IV and oral administration in the setting of IS had been made. However, it is still uncertain if in humans the IV route provides better results for fingolimod. For instance, it has been shown that an IV administration, despite leading to higher maximal concentrations, led to decreased levels of fingolimod’s bioactive metabolite and a less pronounced acute reduction of lymphocytes [77]. Therefore, it seems that the oral route will continue to be the preferred one.

At this point, it is important to point out that inflammation may not be the ultimate villain in ischemia, and the pathophysiological understanding of such biological processes is not always black and white [78]. For instance, several of the so-called “inflammatory cells” migrate into ischemic regions and then acquire functions that are not related to inflammation. Monocytes are an example of this, as they transform into mature phagocytes and assist repairing processes. Similarly, the functions of several immune cell subclasses, such as those of the T regulatory cells, have not been clearly elucidated regarding ischemia [11]. As such, targeting “inflammation” as an over-generalized whole might not be beneficial in the long run, and the interventions need to be carefully planned and targeted. A cautionary tale on this matter is the Enlimomab trial, one of the first attempts to target inflammation in an IS setting. Polymorphonuclear granulocytes are the first that enter ischemic regions and are thought to instigate neuronal damage. Based on this, enlimomab, an antibody against a neutrophil adhesion molecule, was hypothesized to improve prognosis, but instead, patients that received the agent were less likely to have a symptom-free recovery, and even presented higher mortality rates [79].

In the aforementioned study, the safety issue was also raised; patients receiving the antibody presented more adverse effects, namely infections and fever, and this is a serious issue with interventions to the immune system. Even animal studies can show the negative effects of some of the tested drugs; for instance, IFN-b led to decreased weight and a series of hematological alterations in test subjects [45]. In the ongoing clinical trial for the use of IFN-b in IS, patients will need to be premedicated in order to prevent fever, a common side-effect of the regime, which can hinder recovery prospects. Luckily, regarding the treatments that have gathered the most attention, the rate of side-effects does not seem to be higher than in the control groups. The ACTION II trial that led to the discontinuation of the efforts in studying natalizumab in IS at least reported that adverse events (serious or not) and death rates were the same between controls and treated patients, in either dosage of the drug [27]. In its precedent ACTION trial, serious adverse events were of similar frequency between groups, though two of the treated patients died due to complications attributed to the drug [26].

Regarding fingolimod, it has been shown to induce lymphopenia upon its administration, and this reduction in lymphocytes is even used to monitor its action [29]. Fingolimod is associated with cardiovascular side-effects, for example bradycardia [80] and reduced left ventricular function [81], increases the risk of infections, particularly viral, due to the lymphopenia, and may even confer susceptibility towards some malignancy types [82]. However, in the available clinical studies, no serious adverse effects were noted for the treated IS patients [28,29,30], or in a cerebral hemorrhage setting [83]. The lymphopenia can be quickly reversed upon cessation and fingolimod, in the way it is usually administered in IS patients, i.e., for three days at a dosage of 0.5mg, might not suffice to cause serious adverse effects. In any case, a longitudinal observation of IS patients that received fingolimod is advised for future studies, in order to confirm the agent’s safety.

The optimal treatment duration is another unanswered question. The three-day treatment seems to be effective in ameliorating outcomes, and the benefits of fingolimod stem from its action on early processes [28]. A prolonged use may not confer additional benefits, and may even cause harm, in the form of adverse effects, such as serious post-stroke infections, or the hindering of neuronal recovery, which may depend on cellular lines and processes that have been inhibited by the agent. As such, we do not believe that an extended use will indeed prove beneficial, but evidence supporting or rejecting this claim is obviously lacking.

As for the rest of the DMDs that have only been preclinically trialed, IFN-b seems to carry the strongest evidence, both regarding the number of studies, and consensus towards the substance being efficient. One clinical trial is being conducted as of now, and it will be very interesting to see whether in this case, the human results match those of the animal studies. Cop-1 also seems to carry potential, and it is the only DMD with a study involving comorbid animals, with diabetes mellitus in particular. Studies with comorbid animals are also urgently needed for the rest of the DMDs, since it is very rarely that IS occurs in humans without preexisting conditions. Finally, DMF may be another promising candidate, since the available studies have mostly yielded positive results, and it promotes neurogenesis, something of high clinical importance regarding outcomes.

Moving on, MS and IS share several other common elements besides neuroinflammation. Firstly, the genetic background of MS is indisputable [10,84,85]. Recent reports have claimed that MS and IS are also genetically linked [86], with numerous shared genes and similar gene expression levels [87]. One step further than genetics, epigenetics is also similarly altered in both of these entities. For example, the aberrant methylation of DNA is a pathophysiological process thought to play an important part in the development of MS [88], and it has also been highlighted in the setting of IS [89]. The hypermethylation of promoter regions is noted in MS and IS alike, while the promoters of specific genes seem to be targeted in both entities, such as HLA-DRB1 [90,91]. Similarly, CpG islands also present altered methylation patterns in IS and MS [92,93,94]. These common genetic and epigenetic alterations strongly showcase the similarities between MS and IS, and enhance the notion that MS DMDs warrant further research in the setting of IS. Additionally, MS patients are shown to be at greater risk of developing IS [95,96,97], and therefore, the research on whether MS DMDs can ameliorate outcomes of IS gains more importance. It shall be interesting to see in future, studies that have longitudinally followed MS patients under different treatment regimes, and explore how the various DMDs have possibly influenced IS risk or IS outcome. This will considerably add to the existing literature, regarding their potential in improving IS parameters, something that has been little addressed in a few studies that “pretreated” the animals before proceeding with the cerebral ischemia.

## 5. Conclusions

The overlap between IS and MS has raised the expectation of a new era of studiesexploring the use of MS DMDs in cerebral ischemia. Most DMDs have only been involved in animal studies, which have usually presented positive results. So far, the translation of animal studies into clinical trials has provided one “failure”, after natalizumab could not provide benefits, but other efforts concerning fingolimod have been encouraging. If the safety and efficacy of fingolimod can be proven in larger cohorts, it is possible that this agent will be added to the arsenal of drugs promoting recovery after an IS incident. However, many parameters need to be defined before it is eventually applied in standard clinical practice, and many more studies are also needed for the other DMDs’ potential to shine.

## Figures and Tables

**Table 1:** Basic Characteristics and Modes of Action of the Included multiple sclerosis (MS) disease modifying drugs (DMDs).

Substance	Commercial Name, Company	Administration	Mode of Action
Natalizumab	Tysabri, Biogen	Intravenous	Selective inhibition of adhesion molecules, via binding to the α4β1 subunit of human integrins, blocking leukocyte influx into the CNS * [18]
Fingolimod (FTY720)	Gilenya, Novartis	Oral	S1P ** receptor agonist, via functional antagonism influences the trafficking of CD4+ T cells and the migration of lymphocytes into the CNS [19]
Interferon-beta	Rebif, Merck SeronoAvonex, Genesis Pharma	Subcutaneous, intramuscular	Regulation of numerous pathways involved in physiological cellular death and survival, activated after pathogenic injury, inducing the production of many cytokines [20,21]
Glatiramer acetate	Copaxone, Teva	Subcutaneous	Development of protective autoimmunity, activation of immunomodulatory functions of helper and regulatory T cells, shift in the production of cytokines to an anti-inflammatory phenotype, induction of pro-neurogenic factors [22,23]
Teriflunomide	Aubagio, Sanofi-Aventis	Oral	Selective inhibition of dihydro-orotate dehydrogenase, hindering the proliferation of activated B and T lymphocytes, and their migration into the CNS [24]
Dimethyl fumarate	Tecfidera, Biogen	Oral	Alteration in the composition of leukocyte subgroups, shifting their phenotype to anti-inflammatory, induction of T-cell apoptosis, downregulation of adhesion molecules and blocking of leucocyte permeation through the brain–blood barrier (BBB) [25]

* Central nervous system. ** Sphingosine-1-phosphate.

**Table 2:** Brief Summary of Human Studies’ Results on the Use of MS DMDs in Ischemic Stroke (IS).

Author, Year	DMD	Infarct Reduction	Functional Outcome	Adverse Events	Overall
Elkins et al., 2017 [26]	Natalizumab	0	+/0	-	Slightly favors intervention
Elkind et al., 2020 [27]	Natalizumab	NA	-	0	Favors controls
Fu et al., 2014 [28]	Fingolimod	+	+	0	Favors intervention
Zhu et al., 2015 [29]	Fingolimod	+	+	0	Favors intervention
Tian et al., 2018 [30]	Fingolimod	+	+	0	Favors intervention
Liantao et al., 2019 [31]	Fingolimod	+	+	0	Favors intervention

0: no effect/difference between patients and controls, +: positive effect or fewer adverse events, -: negative effect or more adverse events. NA: not assessed.

**Table 3:** Studies Involving Humans, on the Use of Multiple Sclerosis DMDs in IS.

Reference	DMD	Study Design	Results
Elkins et al., 2017 (ACTION) [26]	Natalizumab	Double-blind, phase II 1:1 randomized for 300mg IV natalizumab (*n* = 79) or placebo (*n* = 82) Primary endpoint: change in infarct volume from baseline to day 5 Secondary endpoints: change in infarct volume from baseline to day30, and from 24 h to days 5 and 30; National Institute of Health Stroke Scale (NIHSS) at baseline, 24 h, and at days5 (or discharge), 30, and 90; modified Rankin Scale (mRS) and Barthel Index (BI) at days 5 (or discharge), 30,and 90	With natalizumab: no reduction in infarct volume growth from baseline to day 5 compared with placebo (median absolute growth 28 mL (range −8 to 303) vs. 22 mL (−11 to 328); relative growth ratio 1.09 (90% CI 0.91–1.30, *p* = 0.78) or to day 30 (4 mL (−43 to 121) vs. 4 mL (−28 to 180)); 1.05 ((0.88–1.27), *p* = 0.68), from 24 h to day 5 (8 mL (−30 to 177) vs. 7 mL (−13 to 204)); 1.00 ((0.89–1.12), *p* = 0.49), and from 24h to day 30 (−5 mL (−93 to 81) vs. −5 mL (−48 to 48)); 0.98 ((0.87–1.11), *p* = 0.40), No difference in NIHSS scores (≤1 or ≥8 point improvement) from baseline at 24 h, day 5 (or discharge), day 30 (27 (35%) vs. 36 (44%)); odds ratio 0.69 (90% CI 0.39–1.21, *p* = 0.86), and day 90 (36 (47%) vs. 37 (46%); 1.10 (0.63–1.93), *p* = 0.39). With natalizumab: more patients with mRS scores of 0–1 at day 30 (13 (18%) vs. seven (9%)); odds ratio 2.88 (90% CI 1.20–6.93, *p* = 0.024) and day 90 (18 (25%) vs. 16 (21%); 1.48 (0.74–2.98), *p* = 0.18); and BI score ≥ 95 at day 90 (34 (44%) vs. 26 (33%); 1.91 (1∙07–3∙41), *p* = 0.033); no significant difference at day 5 or 30 (26 (34%) vs. 26 (32%); 1.13 (0.63–2.00), *p* = 0.37). No significant differences in adverse events (AEs) rates (77 (99%) of 78 patients vs. 81 (99%) of 82 patients), serious adverse events (36 (46%) vs. 38 (46%)), and deaths (14 (18%) vs. 13 (16%)); two recorded deaths from events considered relevant to natalizumab
Elkind et al., 2020 (ACTION II) [27]	Natalizumab	Double-blind phase IIb 1:1:1 randomized for 300 mg or 600mg of IV natalizumab or placebo (*n* = 88, 89 and 90 respectively) Assessments at baseline,24 ± 6 h, 5 days, 30 days ± 5 days, and90 ± 5 days; mRS and the BI at 5 days, 30 days ±5 days, and 90 ± 5 days Primary endpoint: composite global measure of functional disability, i.e., mRS 0–1 and BI≥95, at day 90 Secondary outcomes: changes in mRS, BI, Stroke Impact Scale-16 (SIS-16), Montreal Cognitive Assessment (MoCA), and NIHSS scores at the aforementioned timepoints, incidences ofAEs and serious AEs	No efficacy difference between treatment branches (*p* = 0.570): the analysis population then included both treatment windows With natalizumab: smaller possibility of an excellent outcome with either dosage (odds ratio (OR) 0.60; 95% confidence interval 0.39–0.93); no effect of modification by time to treatment or use of thrombolysis/thrombectomy. Global composite good outcome (mRS score:0–2 and BI score ≥ 85) less likely with the treatment (OR (95% CI): 0.53 (0.32–0.87)) No significant differences in favor of natalizumab in the other metrics and scores applied No differences in AE incidence in 300mg, 600mg or placebo (90%, 92%,and 92%, respectively), serious AEs (26%, 33%, and 21% respectively), and deaths (7%, 5%, and 6%, respectively)
Fu et al., 2014 [28]	Fingolimod	Open-label, evaluator-blinded 1:1 randomized, for 0.5mg oral fingolimod for 3 days plus standard treatment, vs. standard treatment only, >4.5h after onset (*n* = 11 each) Assessments: at baseline, at fingolimod administration, and on days 7, 14, 30 and 90, via means of NIHSS, mRS and BI scores	With fingolimod, within the first week: improved neurological function (NIHSS score reduction 4 vs. −1, *p* = 0.0001); reduced infarct growth (9 vs. 27 mL, *p* = 0.0494). With fingolimod, at 90 days: NIHSS scores significantly lower (1.7 ± 0.8 vs. 5.8 ± 1.7, *p* = 0.02), modified BI scores significantly higher (62 ± 11 vs. 87 ± 8, *p* = 0.0049), mRS 0–1 in 73% of the treated, vs. 0% in controls (*p* = 0.009) More beneficial for patients with total or partial anterior circulation occlusion vs. patients with lacunar infarcts (improvement ≥ 4 points on NIHSS in the first week: 80% vs. 0%, *p* = 0.02) No serious AEs noted
Zhu et al., 2015 [29]	Fingolimod	Open label, evaluator-blinded 1:1 randomized for alteplase and 0.5mg oral fingolimod for 3 days (*n* = 22), vs. alteplase only (*n* = 25) Assessments: NIHSS at baseline, upon alteplase administration, on days 1, 7, 14 and 90, and mRS at day 90 Primary outcomes: changes in lesion volume from baseline to day 1, hemorrhage volume at day 1, clinical improvement at day 1 (NIHSS score changes from baseline to day 1) Secondary outcomes: lesion volume growth from day 1 to day 7, clinical improvement from day 1 to day 7 (NIHSS score changes), probability of excellent recovery at day 90 (mRS 0–1)	With fingolimod: reduction in lesion volume (10.1 ± 1.2 vs. 34.3 ± 10.4 mL, *p* = 0.04), smaller hemorrhage volumes (1.2 ± 0.4 vs. 4.4 ± 1.1mL, *p* = 0.01), improved neurological function (NIHSS: 4 (0–8) vs. 2 (−2–8), *p* = 0.02) on day 1; restricted infarct growth (−2.3 ± 2.7 vs. 12.1 ± 3.7 mL, *p* < 0.01), and reduced NIHSS scores (2.5 (0–7) vs. 1 (−4–5), *p* < 0.01) within a week; better recovery at day 90 (mRS 0–1, 73% vs. 32%, *p* < 0.01) No patients with symptomatic hemorrhage on day 1; one patient in the alteplase only group required craniotomy No serious AEs noted
Tian et al., 2018 [30]	Fingolimod	Open-label, blinded endpoint 1:1 randomized to receive alteplase and 0.5mg oral fingolimod for 3 consecutive days or alteplase-alone (*n* = 23 each), 4.5–6h after onset Primary endpoints: reduction in NIHSS in day 1, mRS change at day 90 Secondary outcomes: vessel recanalization, anterograde reperfusion, retrograde reperfusion of collateral flow	With fingolimod: reduction in NIHSS scores at 24 h (4 vs. 0, *p* = 0.004),better outcome at day 90 (mRS = 0–2/3–4/5–6: 57%/34%/9%, vs. 22%/39%/39%, *p* = 0.037);bigger reduction in perfusion lesion (relative median perfusion lesion decrease: 0.7 vs. 0.1, *p* < 0.001), restriction of infarct growth (relative median infarct lesion growth: 1.4 vs. 0.1, *p* < 0.001) by 24 h; smaller relative infarct volume growth between day 7 and24 h (0.2 vs. 0.4, *p* = 0.031); improved anterograde reperfusion of downstream territory (mTICI = 2b–3: 80% vs. 33%, *p* = 0.023) at24 h; prevention of failure of retrograde reperfusion from collateral circulation (University of Calgary pial arterial filling ordinary score = 2 vs. −1, *p* < 0.001) Complete recanalization (mTICI = 2b–3)or sustained collateral circulation (change of pial arterial filling ordinary score ≥0) strongly associated with better outcome (mRS = 0–2) at 90 days (OR = 4.9, 95% CI = 1.0–23.3, *p* = 0.044 No significant difference in symptomatic intracranial hemorrhage (4% vs. 8%) No serious AEs noted
Liantao et al., 2019 [31]	Fingolimod	1:1 randomized for alteplase and fingolimod, or alteplase only (*n* = 45 only) Assessments: NIHSS, BI, mRS scores, at baseline, and days 7, 14, and 90	With fingolimod: no significant difference in NIHSS score, mRS score and BI index at 14 days; significant reduction in NIHSS (mean score 1 vs. 3) and mRS scores (mean score 1 vs. 1.5), and significant increase in BI scores (mean score 100 vs. 80) (*p* < 0.05)at day 90; reduction in infarct volume at day 7 (shown in figures) No difference in AE rates (infections); fewer patients with gastro-intestinal bleeding in the treated group (3 vs. 1)

## Data Availability

No new data were created or analyzed in this study. Data sharing is not applicable to this article.
